# Improving the analysis of composite endpoints in rare disease trials

**DOI:** 10.1186/s13023-018-0819-1

**Published:** 2018-05-22

**Authors:** Martina McMenamin, Anna Berglind, James M. S. Wason

**Affiliations:** 1 MRC Biostatistics Unit, University of Cambridge, Forvie Site, Cambridge, UK; 2 Global Medicines Development, Biometrics and Information Sciences, AstraZeneca, Gothenburg, Sweden; 3 Institute of Health and Society, Newcastle University, Newcastle, UK

**Keywords:** Responder analysis, Composite endpoints, Improving efficiency

## Abstract

**Background:**

Composite endpoints are recommended in rare diseases to increase power and/or to sufficiently capture complexity. Often, they are in the form of responder indices which contain a mixture of continuous and binary components. Analyses of these outcomes typically treat them as binary, thus only using the dichotomisations of continuous components. The augmented binary method offers a more efficient alternative and is therefore especially useful for rare diseases. Previous work has indicated the method may have poorer statistical properties when the sample size is small. Here we investigate small sample properties and implement small sample corrections.

**Methods:**

We re-sample from a previous trial with sample sizes varying from 30 to 80. We apply the standard binary and augmented binary methods and determine the power, type I error rate, coverage and average confidence interval width for each of the estimators. We implement Firth’s adjustment for the binary component models and a small sample variance correction for the generalized estimating equations, applying the small sample adjusted methods to each sub-sample as before for comparison.

**Results:**

For the log-odds treatment effect the power of the augmented binary method is 20-55% compared to 12-20% for the standard binary method. Both methods have approximately nominal type I error rates. The difference in response probabilities exhibit similar power but both unadjusted methods demonstrate type I error rates of 6–8%. The small sample corrected methods have approximately nominal type I error rates. On both scales, the reduction in average confidence interval width when using the adjusted augmented binary method is 17–18%. This is equivalent to requiring a 32% smaller sample size to achieve the same statistical power.

**Conclusions:**

The augmented binary method with small sample corrections provides a substantial improvement for rare disease trials using composite endpoints. We recommend the use of the method for the primary analysis in relevant rare disease trials. We emphasise that the method should be used alongside other efforts in improving the quality of evidence generated from rare disease trials rather than replace them.

**Electronic supplementary material:**

The online version of this article (10.1186/s13023-018-0819-1) contains supplementary material, which is available to authorized users.

## Background

For stakeholders in rare disease communities, it is imperative to keep in mind that rare diseases are far from ‘rare’ for those whose lives they consume. The last few decades have seen a societal shift which recognises this and has resulted in a much greater focus on rare disease research. This is characterised by a surge in patient advocacy groups, a shift in regulation and incentives, increased government funding of rare disease research and advances in technologies to improve international communication between rare disease experts and patients [1]. Despite this, for most rare diseases if treatment options even exist many of them have been approved with very limited evidence. Novel statistical design and analysis methods are needed to make the best use of information provided by studies in rare diseases [2, 3].

One way to maximise information from rare disease trials is to use composite endpoints [4]. These are endpoints which combine a number of individual outcomes in order to assess the effectiveness or efficacy of a treatment. They are typically used in situations where it is difficult to identify a single relevant endpoint to sufficiently capture the change in disease status incited by the treatment. Furthermore, if the components are appropriately chosen, composite endpoints that require an event in only one of the components (a or b or c etc.) may have the ability to improve the power to show a given treatment effect due to the increased number of events [5–7]. These characteristics appeal to rare diseases where many realisations of the diseases are highly variable and availability of the population may be a binding constraint.

Many composite endpoints take the form of responder indices where a binary indicator is formed based on whether the patient has experienced a predefined change in each of the components or not. In particular, in many disease areas the composite is a mixture of continuous and binary components. These endpoints frequently feature in rare autoimmune diseases and rare cancers. Examples of these are presented in Table 1, one of which is the chronic inflammatory disorder Behçet disease. A review of the research performed in this area concludes that evidence continues to be generated from anecdotal case reports rather than randomised trials [8]. As well as those shown in Table 1, any rare cancers using RECIST criteria (Response Evaluation Criteria In Solid Tumors) to define responders and non-responders use endpoints which assume this structure [9].

**Table 1:** Examples of rare diseases which could make use of the augmented binary method

Disease	Example responder endpoint
Primary biliary cholongitis (PBC)	∙ ALP <1.67×ULN
	∙ Total bilirubin < ULN
	∙ ALP decrease ≥ 15%
Behçets disease	∙ Length of principal intestinal
	ulcer compared to size at baseline (%)
	∙ No new lesions
Lupus Nephritis	∙ eGFR no more than 10% below
	preflare value or normal
	∙ Proteinuria UPC ratio < 0.5
	∙Urine sediment: Inactive
	∙ No rescue therapy
Neuroblastoma	∙ < 10mm residual soft tissue at
	primary site
	∙ Complete resolution of MIBG of
	FDG-PET uptake (for MIBG non avid
	tumours) at primary site
Advanced hepatocellular carcinoma	∙ < 20% increase in the sum of the
	longest diameters of target lesions
	∙ No new lesions

*ALP* alkaline phosphatase, *ULN* upper limits of normal, *eGdFR* estimated glomerular filtration rate, *UPC* urinary protein to creatinine, *MIBG* metaiodobenzylguanidine, *FDG-PET* 18-fluorodeoxyglucose positron emission tomography

Analyses of these outcomes typically treat them as binary, thus only using the dichotomisations of continuous components. An alternative in these circumstances is the augmented binary method [10]. This involves jointly modelling the continuous component with the binary component in order to improve the efficiency of estimates by making use of how close patients were to being responders in the continuous component. For a fixed sample size, the method was shown to provide a substantial increase in the power over the standard binary method currently in use, whilst still making inference on the outcome of interest to clinicians. This was illustrated in both solid tumour cancer and rheumatoid arthritis data [10, 11].

Although the method provides substantially more power it also uses more parameters. Some evidence has suggested that it may not be suitable for trials with small samples, perhaps due to issues with asymptotics [10]. We will explore the properties of the augmented binary method in small samples and introduce and implement two small sample corrections from the literature to determine whether we can improve the performance.

If the gains provided by the augmented binary method in common diseases can be realised in smaller samples, this may allow us to gain information from randomised trials that would otherwise not have been possible. This could greatly improve outcomes for many rare disease patients. Further small sample applications of the method include earlier phase 2 trials, or when more doses are of interest but the number of patients are limited.

## Methods

### Data

In order to investigate the properties of the methods in small samples, we will use data from the OSKIRA-1 trial [12]. The trial was a phase III, multicentre, randomised, double-blind, placebo-controlled, parallel-group study investigating the use of fostamatinib in patients with active rheumatoid arthritis. For the purpose of investigating the small sample properties of the methods, we will only make use of two of the three arms in the trial, namely the fostamatinib 100 mg bid for 52 wks arm and the placebo arm.

**Fig. 1:**
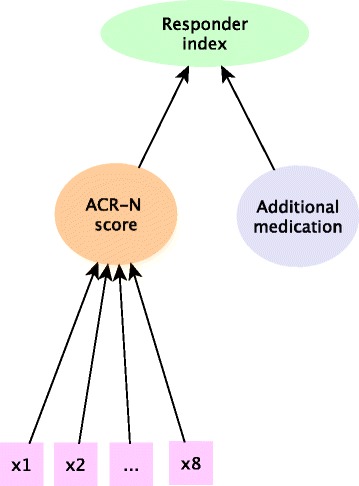
Structure of the responder endpoint in rheumatoid arthritis. For the ACR20 endpoint, the continuous ACR-N score is dichotomised at 20% and combined with the rescue medication indicator to form a binary responder index. X1...X8 denotes the disease activity measures which are combined to form the continuous ACR-N score

A common responder endpoint used in rheumatoid arthritis is the ACR20, in which patients demonstrate clinical response if they achieve a 20% improvement from baseline, as measured by a continuous ACR-N (American College of Rheumatology) score. It is worth noting that the ACR-N score is a percentage change from baseline which is itself a composite combining 7 components but in what follows we will treat this as a single measure, as is the case in practice. The structure of the endpoint is shown in Fig. 1.

A benefit of responder analyses is that we can easily incorporate additional information in the response definition. In the case of rheumatoid arthritis it is common to assign patients to being non-responders in the ACR20 endpoint if they require medications restricted by the protocol or withdraw from the study. Therefore, in order to be a responder to treatment a patient needs to tolerate treatment, must not receive restricted medications and they must demonstrate clinical response. This allows discontinuations of treatment for lack of efficacy or for adverse events to provide meaningful information on the drug effect and translates to estimating the effect of a combination of continuous and binary components.

Other endpoints of interest in rheumatoid arthritis are the ACR50 and ACR70 which dichotomise the ACR-N score at 50% and 70% respectively. We will discuss the findings and conclusions for the ACR20 endpoint in what follows, as this was the primary endpoint in the trial and is the endpoint that is generally used to formally evaluate benefit in the regulatory setting. Results for both the ACR50 and ACR70 endpoints are detailed in the supplementary material (see Additional file 1). These endpoints further characterise the benefit of a treatment by considering different levels of improvement from baseline. Furthermore, they will demonstrate how the methods perform with different response rates.

### Standard binary method

The method currently employed to analyse these endpoints in trials is a logistic regression on the binary indicator for response. We refer to this as the standard binary method.

The odds ratio and confidence interval are obtained directly. We can also obtain predicted probabilities for each patient as if they were treated $\tilde {p}_{i1}$ and not treated $\tilde {p}_{i0}$. This allows us to construct both the difference in response probabilities and the risk ratio. Their corresponding confidence intervals are obtained through the delta method, details of which are provided in the supplementary material (see Additional file 2).

### Augmented binary method

The augmented binary method models the joint distribution of the continuous and binary components at multiple time points by employing factorisation techniques to model each of the components separately. We can then combine these to obtain predicted probabilities for each patient as if they were treated $\tilde {p}_{i1}$ and untreated $\tilde {p}_{i0}$ [10]. It follows that we can obtain the difference in response probabilities, the odds ratio and the risk ratio, as well as their confidence intervals as before (see Additional file 2).

**Fig. 2:**
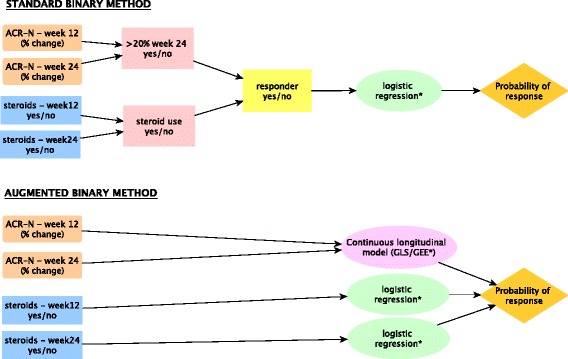
Schematic comparing the stages involved in employing the standard binary and augmented binary methods (*small sample adjustments implemented)

Figure 2 shows a schematic for both the standard binary and augmented binary methods. From this it is clear that the augmented binary method models the components of the composite endpoint directly whereas the standard binary method throws away information before the analysis stage.

Note that we fit the repeated measures models for the continuous component in the augmented binary method using both generalised least squares (GLS) and generalised estimating equations (GEE).

### Binary component adjustment

Albert and Anderson show that when fitting a logistic regression model to small samples, that although the likelihood converges, at least one parameter estimate may be theoretically infinite [13]. This phenomenon is commonly termed ‘perfect separation’ and occurs if the model can perfectly predict the response or if there are more parameters in the model than can be estimated because the data are sparse [14]. Firth provides an alternative to maximum likelihood estimation (MLE) in these circumstances [15]. This involves using penalised maximum likelihood (PML) to correct the mechanism producing the estimate, namely the score equation, rather than the estimate itself.

As maximum likelihood estimates are always biased away from zero in this setting, bias correction therefore involves some degree of shrinkage of the estimate towards this point. This results in the method also reducing the variance, so that bias reduction does not necessarily lead to a substantial loss in power. We will make these adjustments to both the standard binary method and the logit models in the augmented binary method. This can be easily implemented in R using the brglm package [16].

### Continuous component adjustment

It is recognised that when using these methods when the number of clusters, in our case patients, is small that the robust standard error estimates are subject to downward bias, leading to inflated type I errors. We will implement a correction by Morel, Bokossa and Neerchal to inflate the variance estimate when modelling the continuous component using GEE methods [17]. We implement this in R using a modification of the code provided in the geesmv package [18].

The technical details for the models and adjustments are available in the supplementary material (see Additional file 2). The code to implement these in R is also available (see Additional file 3).

### Assessing small sample properties

In order to determine the performance of the unadjusted and adjusted methods, we re-sample from the OSKIRA-1 trial. Employing re-sampling techniques allows us to investigate the properties of the methods under a realistic data structure.

To determine the power we re-sample 5000 replicates for each total sample size between 30 and 80 in increments of 10, which gives a Monte Carlo standard error of 0.3%. To ensure balance we randomly sample half of the total sample size we are interested in from the placebo arm and the other half from the 100 mg arm of the trial. We apply all methods to each sub-sample and record the treatment effect and 95% confidence interval. We do this for both the difference in response probabilities and log-odds estimates of the treatment effect. An estimate of the power is then the proportion of confidence intervals that do not contain zero. By re-sampling, rather than simulating from a known distribution, thinking of this quantity as power implicitly assumes the treatment effect in the trial to be the true treatment effect in the population. To ensure these results agree with the conventional power results, we present the power from a simulated example in the supplementary material (see Additional file 4).

To determine the type I error rate, we permute the treatment labels in the sub-samples in order to remove the association between treatment and outcome. An estimate of the type I error rate is then the proportion of confidence intervals that do not contain zero. The coverage is estimated as the complement of this. Again, to ensure these results agree with when we have simulated under the null, we present an additional simulated example in the supplementary material. The median width of the confidence intervals and the average treatment effect for both methods are also presented in the supplementary material (see Additional file 5).

The unadjusted methods to be applied are the standard binary method, the augmented binary method with GLS and the augmented binary method with GEE. The adjustments refer to the standard binary method fitted with PML, the augmented binary method with GLS and PML and the augmented binary method with the GEE variance correction and PML.

## Results

### Log-odds scale

**Fig. 3:**
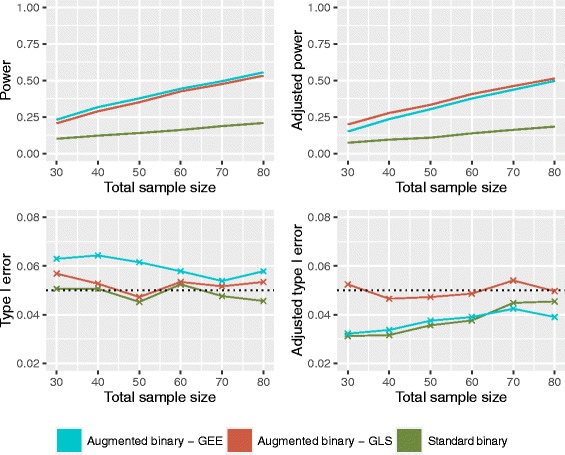
Operating characteristics of the unadjusted (left) and adjusted (right) standard binary and augmented binary methods on the log-odds scale

The power and type I error rates for the unadjusted and adjusted methods are shown in Fig. 3. The unadjusted augmented binary method provides higher power than the standard binary method for all sample sizes. The type I error rate of both methods is approximately 5%. Implementing the firth adjustment in the augmented binary method with GLS makes negligible difference to the power or type I error rate. In the adjusted augmented binary method with GEE, the type I error rate drops to 3–4%. Differences between the GLS and GEE estimators diminish with increasing sample size. The standard binary method experiences a substantial drop in type I error rate when the Firth correction is implemented.

### Probability scale

**Fig. 4:**
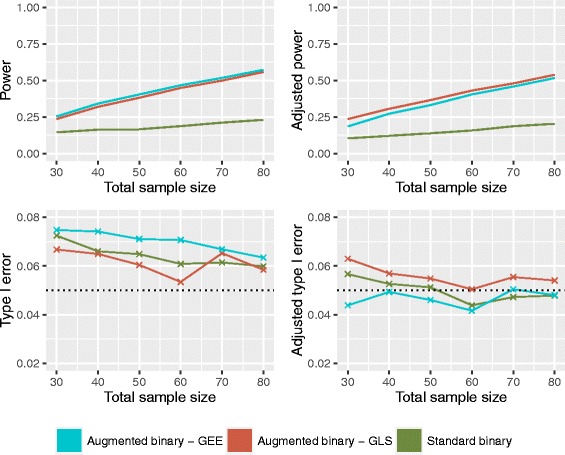
Operating characteristics of the unadjusted (left) and adjusted (right) standard binary and augmented binary methods on the probability scale

Figure 4 shows the power and type I error rates for the difference in response probabilities. The power is similar to the log-odds case however both methods experience an inflation in type I error rate. Implementing the correction in the GLS augmented binary method results in a small improvement in the type I error rate with no power lost. GEE adjustments result in an average reduction in type I error of approximately 2.5% but the power drops to below that of the adjusted GLS. Again, differences in GLS and GEE diminish as the sample size increases. The adjustment for the standard binary reduces the type I error rate from 7% to approximately 5% however the power is below 20% for all sample sizes investigated.

Table 2 shows the average reduction in confidence interval width for the adjusted methods on both scales. We compare the standard binary with both implementations of the augmented binary method. We see from this that the augmented binary method with GLS offers the most precision. This translates to the adjusted augmented binary method requiring a 32% smaller sample size than what would be required for the adjusted standard binary method.

**Table 2:** Comparison in average confidence interval width for the small sample adjusted methods on the log-odds and probability scales

Comparison	Average reduction in C.I. width (%)	Reduction in required sample size (%)
Log-odds		
Standard binary vs Augmented binary (GLS)	17.4	31.8
Standard binary vs Augmented binary (GEE)	11.2	21.1
Difference in response probabilities		
Standard binary vs Augmented binary (GLS)	17.6	32.1
Standard binary vs Augmented binary (GEE)	12.3	23.1

C.I. confidence interval

To better understand the benefit of the small sample corrections it is useful to interpret the proportion of cases experiencing perfect separation alongside the average width of the confidence intervals. Table 3 shows the percentage of the 5000 sub-samples with confidence intervals for the difference in response probabilities larger than 1. This is shown for each method at each sample size. This would suggest that the corrections are most beneficial when N <60.

**Table 3:** Percentage of cases experiencing extremely large variance due to perfect separation on probability scale (confidence interval for difference > 1)

	Standard binary		Augmented binary (GLS)		Augmented binary (GEE)	
N	Unadjusted	Adjusted	Unadjusted	Adjusted	Unadjusted	Adjusted
30	0.00	0.00	10.9	0.00	10.6	0.24
40	0.00	0.00	3.71	0.00	3.81	0.02
50	0.00	0.00	1.11	0.00	1.20	0.00
60	0.00	0.00	0.24	0.00	0.30	0.00
70	0.00	0.00	0.04	0.00	0.08	0.00
80	0.00	0.00	0.00	0.00	0.00	0.00

### Simulated example

Although re-sampling is beneficial as it details performance information under realistic data structures, the findings may be enriched by considering an example from a known distribution. We firstly set the probability of response equal to 0.470 in the treatment arm and 0.336 in the placebo arm, similar to the OSKIRA-1 study. Secondly, we simulate under the null where the probability of response equals 0.336 in both arms. We investigate power, type I error rate, average treatment effect estimates and average confidence interval width for the small sample adjusted binary and augmented binary methods. The results are presented in the supplementary material (see Additional file 4).

In summary, our comparative findings from the re-sampling are supported, in that the augmented binary method offers higher power and precision with a reduction in required sample size of approximately 38%. The augmented binary method has nominal type I error rate, which is consistent with the re-sampling results. However, the type I error for the adjusted standard binary method is 6.8–8.1%, which is higher than the type I error rates found from re-sampling. The absolute power estimates for both methods also differ from those in the re-sampling results, however the comparative conclusions are the same. The methods provide approximately equal treatment effect estimates. A simulated example dataset is included should readers wish to fit the models (see Additional file 6).

## Discussion

In this paper we have explored the small sample properties of the standard binary and augmented binary methods and proposed adjustments to improve them. It would appear that the increased efficiency of the augmented binary method does indeed translate to a small sample setting. The method performs better on the log-odds scale, where normality assumptions made when employing the delta method are best satisfied. These assumptions are more questionable when working with samples of this size on the probability scale, which is partly reflected in the differences in inflation present.

As rare disease trials are restricted in their capacity to detect treatment effects both because of small studies and few studies running in any given disease, it follows that maximising power within a single study is perhaps even more crucial than in more common diseases. Consequently, we recommend the use of the augmented binary method as the primary analysis method in trials of rare diseases using these endpoints.

When implementing the augmented binary method in rare disease trials, we recommend the use of the Firth adjustment for the logit models as it reduces the bias and variance of the estimates. This is especially valuable in this setting due to the restrictive nature of sample size. For the continuous component, we recommend the GLS estimator. As well as offering the largest power and precision, GLS methods make more realistic assumptions about the mechanism for missing responses, namely that they are missing at random rather than missing completely at random. Moreover they experience fewer convergence issues in very small samples.

We have previously acknowledged the potential utility of composite endpoints in rare diseases, however guidance must be followed in order to ensure valid and meaningful implementation in clinical trials [5]. Composite endpoints should be coherent, in that the components are measuring the same underlying pathophysiologic process. However, the components should not be so closely related that the patient is likely to experience all of them, hence making the combined endpoint redundant [19]. The magnitude of the gains from adopting a composite endpoint depends on the correlation between components, the direction of treatment effect in each component and hence the patient responder rates. It is therefore crucial for interpretation that effects are reported on individual components as secondary results. Binary components of the composite can be analysed with standard binary methods. Dichotomised continuous components of the composite may be analysed with standard binary methods, a continuous test or by testing the dichotomised component whilst making use of the continuous information, a technique similar to the augmented binary approach and originally proposed by Suissa [20]. This may be preferable to maintain the clinical definition of the component whilst improving the power.

It is useful to consider further the role of response rate in the composite endpoint on the operating characteristics of interest. The ACR50 and ACR70 results presented in the supplementary material indicate that power and type I error are highly dependant on responder rates and treatment effect scales (see Additional file 1). For the standard binary method, the results show deflations in the type I error rate on the log-odds scale and inflations on the probability scale, with type I error rates ranging from 0 to 10%. This is likely to be due to logistic regression methods having poorly estimated standard errors when there are few events per parameter, as is the case for the ACR50 and ACR70 endpoints [21]. Overall, the augmented binary method shows fewer deviations from nominal type I error rates whilst exhibiting increased power over the standard binary method in every scenario investigated.

The findings from the simulated example in the supplementary material further reiterate these problems with type I error rate control in the standard binary method. As the type I error rate is more stable for the augmented binary method both in the re-sampling and the simulated example, we would suggest that it is perhaps more robust in the rare disease setting than logistic regression methods.

Although it is recognised that novel methods developed for use in rare diseases may be of more immediate utility than in common diseases, some resistance to implementing the augmented binary method in real rare disease trials may be experienced due to its increased complexity. To assist with this we supply full R code for all unadjusted and adjusted versions of the method. It is of paramount importance that the efficiency gains provided by this method are not used as a substitute for other important efforts and considerations undertaken when running rare disease trials. That is, the method should be used to complement efforts in establishing international, multi-centre trials with maximum feasible enrolment periods, alongside other achievable strategies to increase sample size; not to replace them.

There are some limitations in what we have presented. We have only investigated the performance of the method in small samples in relation to the rheumatoid arthritis endpoint. Similar procedures may be carried out in other data sets and the methods applied directly to rare disease data, to ensure these gains are always experienced across a range of responder indices and response rates. Moreover, due to the increased number of parameters, the augmented binary method starts to experience some problems when we reduce the total sample size to n=20. This is unlikely to be a problem in practice, as a randomised trial as small as that would be unusual. If required, it may be possible to make further assumptions in order to reduce the number of parameters to be estimated.

Our future work aims to improve the uptake of the augmented binary method in rare disease trials by developing methods for performing power calculations. This would overcome using the approximation that, for fixed power, the average gains equate to reducing the required sample size by at least 32%. A further extension on which we are currently working is developing joint modelling methods for the instance when the composite is a more complicated combination of outcomes, namely multiple continuous, ordinal and binary components. We expect these methods to exhibit even larger efficiency gains due to using information in multiple continuous and ordinal components. This will provide the potential to improve even further the frequency and quality of evidence generated in many rare disease areas.

## Conclusion

In rare diseases where there are few or no available treatments and limited opportunity to test emerging new treatments, the power to detect an effective treatment is of critical importance. The augmented binary method with small sample adjustments offers a substantial improvement for trials in these populations over methods currently being used, which throw away valuable information. We recommend the use of the augmented binary method in relevant rare disease trials using composite endpoints and supply R code to assist with the implementation.

## Additional files

Additional file 1 Results for the power and type I error rates of the ACR50 and ACR70 endpoints. (PDF 23 kb)

Additional file 2 Notation, models and small sample corrections. Technical detail for the models and small sample corrections. (PDF 92 kb)

Additional file 3 R code to fit methods and small sample adjustments. (ZIP 7 kb)

Additional file 4 Results for simulated examples from a known distribution. (PDF 52 kb)

Additional file 5 Median confidence interval widths and average treatment effect estimates from re-sampling. (PDF 23 kb)

Additional file 6 Simulated example dataset. (CSV 7 kb)

## Abbreviations

ACR: American college of rheumatology
ALP: Alkaline phosphatase
eGFR: Estimated glomerular filtration rate
FDG-PET: 18-fluorodeoxyglucose positron emission tomography
GEE: Generalised estimating equations
GLS: Generalised least squares
MIBG: Metaiodobenzylguanidine
MLE: Maximum likelihood estimation
RECIST: Response evaluation criteria in solid tumors
PML: Penalised maximum likelihood
ULN: Upper limits of normal
UPC: Urinary protein to creatinine
