# Supplementary material for: Improving the analysis of composite endpoints in rare disease trials
Source: Orphanet J Rare Dis. 2018 May 22;13:81. doi: 10.1186/s13023-018-0819-1 (PMC5964664; doi:10.1186/s13023-018-0819-1)
Supplement: Supplementary file 1 — Results for the power and type I error rates of the ACR50 and ACR70 endpoints. (PDF 23 kb) [file 13023_2018_819_MOESM1_ESM.pdf]

Table 1: Type I error of the log-odds ACR50 response in standard binary and augmented binary methods in 5000 sub-samples

	Type I error			Small sample adjusted type I error		
Total sample size	Standard binary	Augmented binary (GLS)	Augmented binary (GEE)	Standard binary	Augmented binary (GLS, PML)	Augmented binary (GEE adj, PML)
30	0.005	0.059	0.069	0.005	0.054	0.035
40	0.011	0.058	0.068	0.010	0.048	0.040
50	0.019	0.053	0.063	0.014	0.056	0.044
60	0.025	0.055	0.060	0.020	0.052	0.041
70	0.034	0.057	0.059	0.025	0.050	0.044
80	0.034	0.056	0.059	0.029	0.053	0.050

GLS generalised least squares, GEE generalised estimating equations, PML penalised maximum likelihood, GEE adj GEE small sample adjustment

Table 2: Power of the log-odds ACR50 response in standard binary and augmented binary methods in 5000 sub-samples

	Power			Small sample adjusted power		
Total sample size	Standard binary	Augmented binary (GLS)	Augmented binary (GEE)	Standard binary	Augmented binary (GLS with PML)	Augmented binary (GEE adj, PML)
30	0.022	0.196	0.232	0.034	0.208	0.150
40	0.061	0.263	0.298	0.073	0.265	0.216
50	0.133	0.324	0.359	0.142	0.321	0.279
60	0.193	0.381	0.407	0.207	0.378	0.339
70	0.269	0.436	0.459	0.277	0.433	0.400
80	0.357	0.493	0.512	0.348	0.490	0.460

GLS generalised least squares, GEE generalised estimating equations, PML penalised maximum likelihood, GEE adj GEE small sample adjustment

Table 3: Type I error of the ACR50 difference in response probabilities in standard binary and augmented binary methods in 5000 sub-samples

	Type I error			Small sample adjusted type I error		
Total sample size	Standard binary	Augmented binary (GLS)	Augmented binary (GEE)	Standard binary	Augmented binary (GLS with PML)	Augmented binary (GEE adj,PML)
30	0.091	0.054	0.065	0.039	0.051	0.031
40	0.065	0.059	0.066	0.045	0.054	0.038
50	0.067	0.055	0.061	0.042	0.054	0.035
60	0.055	0.061	0.062	0.047	0.052	0.046
70	0.054	0.051	0.060	0.051	0.053	0.044
80	0.064	0.049	0.061	0.045	0.058	0.044

GLS generalised least squares, GEE generalised estimating equations, PML penalised maximum likelihood, GEE adj GEE small sample adjustment

Table 4: Power of the ACR50 difference in response probabilities in standard binary and augmented binary methods in 5000 sub-samples

	Power			Small sample adjusted power		
Total sample size	Standard binary	Augmented binary (GLS)	Augmented binary (GEE)	Standard binary	Augmented binary (GLS with PML)	Augmented binary (GEE adj, PML)
30	0.243	0.202	0.208	0.157	0.211	0.133
40	0.273	0.270	0.284	0.224	0.272	0.199
50	0.318	0.334	0.344	0.274	0.329	0.264
60	0.363	0.395	0.394	0.314	0.385	0.324
70	0.415	0.441	0.444	0.370	0.436	0.387
80	0.474	0.500	0.498	0.432	0.496	0.446

GLS generalised least squares, GEE generalised estimating equations, PML penalised maximum likelihood, GEE adj GEE small sample adjustment

Table 5: Type I error of the log-odds ACR70 response in standard binary and augmented binary methods in 5000 sub-samples

Total sample size	Type I error			Small sample adjusted type I error		
	Standard binary	Augmented binary (GLS)	Augmented binary (GEE)	Standard binary	Augmented binary (GLS with PML)	Augmented binary (GEE with PML)
30	0.000	0.063	0.074	0.000	0.056	0.036
40	0.000	0.061	0.068	0.000	0.054	0.041
50	0.000	0.052	0.071	0.000	0.058	0.045
60	0.000	0.056	0.064	0.000	0.058	0.038
70	0.000	0.048	0.058	0.002	0.058	0.046
80	0.002	0.055	0.055	0.002	0.053	0.046

GLS generalised least squares, GEE generalised estimating equations, PML penalised maximum likelihood, GEE adj GEE small sample adjustment

Table 6: Power of the log-odds ACR70 response in standard binary and augmented binary methods in 5000 sub-samples

Total sample size	Power			Small sample adjusted power		
	Standard binary	Augmented binary (GLS)	Augmented binary (GEE)	Standard binary	Augmented binary (GLS with PML)	Augmented binary (GEE with PML)
30	0.000	0.191	0.229	0.000	0.205	0.142
40	0.000	0.247	0.284	0.000	0.253	0.199
50	0.001	0.307	0.340	0.004	0.308	0.258
60	0.003	0.352	0.378	0.007	0.350	0.312
70	0.007	0.411	0.433	0.021	0.411	0.370
80	0.015	0.460	0.478	0.036	0.460	0.424

GLS generalised least squares, GEE generalised estimating equations, PML penalised maximum likelihood, GEE adj GEE small sample adjustment

Table 7: Type I error of the ACR70 difference in response probabilities in standard binary and augmented binary methods in 5000 sub-samples

	Type I error			Small sample adjusted type I error		
Total sample size	Standard binary	Augmented binary (GLS)	Augmented binary (GEE)	Standard binary	Augmented binary (GLS with PML)	Augmented binary (GEE with PML)
30	0.056	0.029	0.031	0.006	0.030	0.008
40	0.040	0.032	0.039	0.013	0.034	0.010
50	0.032	0.031	0.037	0.020	0.034	0.015
60	0.041	0.034	0.041	0.022	0.036	0.022
70	0.049	0.040	0.040	0.020	0.045	0.025
80	0.048	0.045	0.039	0.026	0.040	0.031

GLS generalised least squares, GEE generalised estimating equations, PML penalised maximum likelihood, GEE adj GEE small sample adjustment

Table 8: Power of the ACR70 difference in response probabilities in standard binary and augmented binary methods in 5000 sub-samples

	Power			Small sample adjusted power		
Total sample size	Standard binary	Augmented binary (GLS)	Augmented binary (GEE)	Standard binary	Augmented binary (GLS with PML)	Augmented binary (GEE with PML)
30	0.108	0.118	0.110	0.029	0.120	0.034
40	0.112	0.173	0.165	0.065	0.174	0.077
50	0.136	0.243	0.228	0.095	0.240	0.140
60	0.185	0.304	0.281	0.128	0.301	0.206
70	0.250	0.366	0.345	0.156	0.363	0.269
80	0.288	0.417	0.399	0.184	0.415	0.331

GLS generalised least squares, GEE generalised estimating equations, PML penalised maximum likelihood, GEE adj GEE small sample adjustment
