# Supplementary material for: Improving the analysis of composite endpoints in rare disease trials
Source: Orphanet J Rare Dis. 2018 May 22;13:81. doi: 10.1186/s13023-018-0819-1 (PMC5964664; doi:10.1186/s13023-018-0819-1)
Supplement: Supplementary file 2 — Notation, models and small sample corrections. Technical detail for the models and small sample corrections. (PDF 92 kb) [file 13023_2018_819_MOESM2_ESM.pdf]

MODELS AND SMALL SAMPLE ADJUSTMENTS

NOTATION

Suppose we have i patients, with $i \in \{1, \dots, n\}$. Let $T_i \in \{1,2\}$ indicate the treatment arm of patient i. The baseline ACR-N score is y_{i0}, with Y_{i1}, Y_{i2} denoting the continuous ACR-N scores at the week 12 visit and week 24 visit respectively. F_{i1} is an indicator variable taking a value equal to 1 if the patient discontinues treatment or requires rescue medication before the week 12 visit. F_{i2} is the corresponding indicator for the period between the week 12 and week 24 visit. S_i is then a binary variable indicating whether or not patient i was a responder. For the ACR20 endpoint, $S_i = 1$ if $Y_{i2} \geqslant 20$ and $F_{i1} = F_{i2} = 0$.

STANDARD BINARY METHOD

The standard binary method is a logistic regression on the binary indicator S_i.

$$logit\left(P\left(S_i = 1 | T_i, y_{i0}\right)\right) = \alpha + \beta T_i + \gamma y_{i0} \tag{1}$$

This provides us with maximum likelihood estimates $\hat{\theta}_{SB} = \{\alpha, \beta, \gamma\}$ and $Cov(\hat{\theta}_{SB})$. From this we can obtain a fitted probability of response for each patient i as if they were treated with the experimental treatment $\tilde{p}_{i1}$ and the control treatment $\tilde{p}_{i0}$.

From this we can then can construct various quantities of interest:

1. Difference in Response Probabilities

$$\tilde{\delta}_1 = \frac{\sum_{i=1}^{n} \tilde{p}_{i1} - \sum_{i=1}^{n} \tilde{p}_{i0}}{n} \tag{2}$$

2. Risk ratio

$$\tilde{\delta}_2 = \frac{\sum_{i=1}^{n} \tilde{p}_{i1}}{\sum_{i=1}^{n} \tilde{p}_{i0}} \tag{3}$$

3. Odds ratio

$$\tilde{\delta}_3 = \frac{\left(\frac{\sum_{i=1}^{n} \tilde{p}_{i1}}{n - \sum_{i=1}^{n} \tilde{p}_{i1}}\right)}{\left(\frac{\sum_{i=1}^{n} \tilde{p}_{i0}}{n - \sum_{i=1}^{n} \tilde{p}_{i0}}\right)} \tag{4}$$

Confidence intervals for these treatment effect estimates can be constructed by obtaining standard error estimates through the delta method. This requires the covariance matrix of the maximum likelihood estimates Cov$(\hat{\theta}_{SB})$ and the vector of partial derivatives of $\tilde{\delta}$ with respect to each of the parameter estimates, $''\tilde{\delta}$.

For example, the variance of $\tilde{\delta}_1$:

$$Var(\tilde{\delta}_1) = (''\tilde{\delta}_1)^T Cov(\hat{\theta}_{SB})(''\tilde{\delta}_1) \tag{5}$$

AUGMENTED BINARY METHOD

The augmented binary method models the joint distribution of $(Y_1, Y_2, F_1, F_2)|T, Y_0$ by employing factorisation techniques to model each of the components separately, as shown by the equations below.

$$Y_{ij} = \alpha + \beta_1 T_i I\{j=1\} + \beta_2 T_i I\{j=2\} + \gamma y_{i0} + \delta_j + \varepsilon_{ij}$$
$$(\varepsilon_{i1}, \varepsilon_{i2}|T_i, y_{i0}) \sim N\left((0,0), \begin{bmatrix} \sigma_1^2 & \rho\sigma_1\sigma_2 \\ \rho\sigma_1\sigma_2 & \sigma_2^2 \end{bmatrix}\right) \quad (6)$$

$$logit\big(P(F_{i1}=1|T_i, y_{i0}, Y_{i1}, Y_{i2})\big) = \alpha_{F1} + \beta_{F1} T_i + \gamma_{F1} y_{i0} \quad (7)$$

$$logit\big(P(F_{i2}=1|F_{i1}=0, T_i, y_{i0}, Y_{i1}, Y_{i2})\big) = \alpha_{F2} + \beta_{F2} T_i + \gamma_{F2} Y_{i1} \quad (8)$$

We fit repeated measures models using both generalised least squares (GLS) and generalised estimating equations (GEE) to the continuous component. GLS estimates the variance-covariance matrix using restricted maximum likelihood methods and GEE makes use of robust variance estimation techniques.

After fitting these models and obtaining maximum likelihood estimates $\hat{\theta}_{AB} = \{\hat{\alpha}, \hat{\beta}_1, \hat{\beta}_2, \hat{\gamma}, \hat{\delta}_1, \hat{\delta}_2, \hat{\alpha_{F1}}, \hat{\beta_{F1}}, \hat{\gamma_{F1}}, \hat{\alpha_{F2}}, \hat{\beta_{F2}}, \hat{\gamma_{F2}}\}$, we can obtain the overall probability in response in each arm. For patient *i*, the probability of response in the ACR20 endpoint is:

$$\begin{aligned}
&P(Y_{i2} \geqslant 20, F_{i1} = F_{i2} = 0|T_i, y_{i0}) \\
&= \int_{-\infty}^{\infty}\int_{-\infty}^{\infty} P(Y_{i2} \geqslant 20, F_{i1} = F_{i2} = 0|T_i, y_{i0}, Y_{i1} = y_{i1}, Y_{i2} = y_{i2}) f_{y_{i1},y_{i2}}(y_{i1}, y_{i2}; T_i, y_{i0})\, dy_{i2}\, dy_{i1} \\
&= \int_{-\infty}^{\infty}\int_{20}^{\infty} P(F_{i1} = F_{i2} = 0|T_i, y_{i0}, Y_{i1} = y_{i1}, Y_{i2} = y_{i2}) f_{y_{i1},y_{i2}}(y_{i1}, y_{i2}; T_i, y_{i0})\, dy_{i2}\, dy_{i1} \\
&= \int_{-\infty}^{\infty}\int_{20}^{\infty} P(F_{i2} = 0|F_{i1} = 0, T_i, y_{i0}, Y_{i1} = y_{i1}) P(F_{i1} = 0|T_i, y_{i0}, Y_{i1} = y_{i1}) f_{y_{i1},y_{i2}}(y_{i1}, y_{i2}; T_i, y_{i0})\, dy_{i2}\, dy_{i1}
\end{aligned}$$

Again, we can obtain a fitted probability of response for each patient *i* as if they were treated with the experimental treatment $\tilde{p}_{i1}$ and the control treatment $\tilde{p}_{i0}$. Treatment effect estimates and confidence intervals are constructed as before, where $Cov(\hat{\theta}_{AB})$ is as shown in equation (9).

$$Cov(\hat{\theta}_{AB}) = \begin{pmatrix} Cov(\hat{\alpha}, \hat{\beta}_1, \hat{\beta}_2, \hat{\gamma}, \hat{\delta}_1, \hat{\delta}_2) & 0 & 0 \\ 0 & Cov(\hat{\alpha_{F1}}, \hat{\beta_{F1}}, \hat{\gamma_{F1}}) & 0 \\ 0 & 0 & Cov(\hat{\alpha_{F2}}, \hat{\beta_{F2}}, \hat{\gamma_{F2}}) \end{pmatrix} \quad (9)$$

BINARY COMPONENT ADJUSTMENT

The penalised likelihood is shown below, where $L(\theta)$ is the usual likelihood function for a logit model and $I(\theta)$ is the information matrix.

$$L^*(\theta) = L(\theta)|I(\theta)|^{\frac{1}{2}} \quad (10)$$

CONTINUOUS COMPONENT ADJUSTMENT

The standard robust sandwich covariance estimator is shown in equation 11.

$$V_{sandwich} = (\sum_{i=1}^{n} D_i V_i^{-1} D_i)^{-1} (\sum_{i=1}^{n} D_i V_i^{-1} Co\widehat{v}(Y_i) V_i^{-1} D_i)(\sum_{i=1}^{n} D_i V_i^{-1} D_i)^{-1} \quad (11)$$

where:
$D_i = \frac{\partial \mu_i}{\partial \beta}$
μ_i is the vector of mean responses
β the parameter vector
V_i is the working variance-covariance matrix for Y_i
$Co\widehat{v}(Y_i) = (Y_i - \hat{\mu}_i)(Y_i - \hat{\mu}_i)'$.

The small sample adjusted variance estimator is shown in equation 12.

$$V_{MBN} = \left(\sum_{i=1}^{n} D_i V_i^{-1} D_i\right)^{-1} \left(\sum_{i=1}^{n} D_i V_i^{-1} \left(kCo\widehat{v}(Y_i) + \delta_m \xi V_i\right) V_i^{-1} D_i\right) \left(\sum_{i=1}^{n} D_i V_i^{-1} D_i\right)^{-1} \quad (12)$$

where:
$k = \frac{N-1}{N-p} \frac{n}{n-1}$
p is the number of parameters
N is the total number of observations
$$\delta_m = \begin{cases} \frac{p}{n-p}, & \text{if n>3p} \\ \frac{1}{2}, & \text{otherwise} \end{cases}$$
$$\xi = max\left(1, \frac{trace\left((\sum_{i=1}^{n} D_i V_i^{-1} D_i)^{-1} (\sum_{i=1}^{n} D_i V_i^{-1} Cov(Y_i) V_i^{-1} D_i)\right)}{p}\right)$$
