# Supplementary material for: Improving the analysis of composite endpoints in rare disease trials
Source: Orphanet J Rare Dis. 2018 May 22;13:81. doi: 10.1186/s13023-018-0819-1 (PMC5964664; doi:10.1186/s13023-018-0819-1)
Supplement: Supplementary file 4 — Results for simulated examples from a known distribution. (PDF 52 kb) [file 13023_2018_819_MOESM4_ESM.pdf]

SIMULATED EXAMPLE

NOTATION

Suppose we have i patients, with $i \in \{1,...,n\}$. Let $T_i \in \{1,2\}$ indicate the treatment arm of patient i. The baseline ACR-N score is y_{i0}, with Y_{i1}, Y_{i2} denoting the continuous ACR-N scores at the week 12 visit and week 24 visit respectively. F_{i1} is an indicator variable taking a value equal to 1 if the patient discontinues treatment or requires rescue medication before the week 12 visit. F_{i2} is the corresponding indicator for the period between the week 12 and week 24 visit. S_i is then a binary variable indicating whether or not patient i was a responder. For the ACR20 endpoint, $S_i = 1$ if $Y_{i2} \geqslant 20$ and $F_{i1} = F_{i2} = 0$.

MODEL

$$\begin{aligned} Y_{ij} &= \alpha + \beta_1 T_i I\{j=1\} + \beta_2 T_i I\{j=2\} + \gamma y_{i0} + \delta_j + \varepsilon_{ij} \\ (\varepsilon_{i1}, \varepsilon_{i2} | T_i, y_{i0}) &\sim N\left((0,0), \begin{bmatrix} \sigma_1^2 & \rho\sigma_1\sigma_2 \\ \rho\sigma_1\sigma_2 & \sigma_2^2 \end{bmatrix}\right) \end{aligned} \tag{1}$$

$$logit\big(P(F_{i1} = 1 | T_i, y_{i0}, Y_{i1}, Y_{i2})\big) = \alpha_{F1} + \beta_{F1} T_i + \gamma_{F1} y_{i0} \tag{2}$$

$$logit\big(P(F_{i2} = 1 | F_{i1} = 0, T_i, y_{i0}, Y_{i1}, Y_{i2})\big) = \alpha_{F2} + \beta_{F2} T_i + \gamma_{F2} Y_{i1} \tag{3}$$

where $y_{i0} \sim N(5.8, 0.8)$ as in the OSKIRA-1 trial.

We investigate the power and type I error rate for the small sample adjusted measures for the difference in response probability estimator, as shown below.

$$\tilde{\delta}_1 = \frac{\sum_{i=1}^{n} \tilde{p}_{i1} - \sum_{i=1}^{n} \tilde{p}_{i0}}{n} \tag{4}$$

where $\tilde{p}_{i1}$ and $\tilde{p}_{i0}$ are the fitted probabilities of response for patient i on the experimental treatment and the control treatment respectively.

RESULTS

Table 1: Power and average confidence interval width in ACR20 response in the small sample adjusted standard binary and augmented binary methods in 5000 simulations

Total sample	$\tilde{\delta}_1$ (S.D.)		Power		Average CI width		Sample size
size	Bin	Aug bin	Bin	Aug bin	Bin	Aug bin	reduction (%)
30	0.128 (0.167)	0.130 (0.121)	0.145	0.172	0.630	0.496	38.0
40	0.132 (0.145)	0.133 (0.106)	0.179	0.226	0.550	0.431	38.6
50	0.138 (0.129)	0.135 (0.097)	0.213	0.278	0.493	0.386	38.7
60	0.137 (0.120)	0.136 (0.088)	0.240	0.329	0.452	0.353	39.0
70	0.135 (0.113)	0.136 (0.083)	0.269	0.367	0.419	0.328	38.7
80	0.138 (0.103)	0.138 (0.077)	0.293	0.425	0.392	0.306	39.1

$\alpha = -15, \beta_1 = 2.5, \beta_2 = 2, \gamma = 4.1, \delta_1 = 6, \delta_2 = 12, \sigma_1 = 1, \sigma_2 = 1, \rho = 0.6, \alpha_{F1} = -3.8, \beta_{F1} = -0.1, \gamma_{F1} = 0.4, \alpha_{F2} = -0.8, \beta_{F2} = -0.08, \gamma_{F2} = -0.008, \tilde{\delta}_1 \approx 0.134$

Table 2: Type I error rate and average confidence interval width in ACR20 response in the small sample adjusted standard binary and augmented binary methods in 5000 simulations

Total sample	$\tilde{\delta}_1$ (S.D.)		Type I error		Average CI width	
size	Bin	Aug bin	Bin	Aug bin	Bin	Aug bin
30	0.002 (0.157)	0.001 (0.102)	0.068	0.047	0.596	0.426
40	-0.001 (0.143)	0.001 (0.092)	0.080	0.047	0.517	0.370
50	0.000 (0.128)	-0.002 (0.081)	0.081	0.044	0.465	0.332
60	-0.001 (0.118)	0.000 (0.075)	0.079	0.043	0.425	0.303
70	-0.001 (0.107)	0.000 (0.070)	0.073	0.043	0.394	0.282
80	0.000 (0.104)	0.000 (0.065)	0.081	0.049	0.369	0.263

$\alpha = -15, \beta_1 = 0, \beta_2 = 0, \gamma = 4.1, \delta_1 = 6, \delta_2 = 12, \sigma_1 = 1, \sigma_2 = 1, \rho = 0.6, \alpha_{F1} = -3.8, \beta_{F1} = 0, \gamma_{F1} = 0.4, \alpha_{F2} = -0.8, \beta_{F2} = 0, \gamma_{F2} = -0.008, \tilde{\delta}_1 = 0$
