# Supplementary material for: Improving the analysis of composite endpoints in rare disease trials
Source: Orphanet J Rare Dis. 2018 May 22;13:81. doi: 10.1186/s13023-018-0819-1 (PMC5964664; doi:10.1186/s13023-018-0819-1)
Supplement: Supplementary file 5 — Median confidence interval widths and average treatment effect estimates from re-sampling. (PDF 23 kb) [file 13023_2018_819_MOESM5_ESM.pdf]

Table 1: Median width (S.D.) of confidence intervals of the standard binary and augmented binary methods for the log-odds treatment effect

	Standard binary		Augmented binary (GLS)		Augmented binary (GEE)	
N	Unadjusted	Adjusted	Unadjusted	Adjusted	Unadjusted	Adjusted
30	3.019 (431.8)	3.000 (0.236)	2.509 (1390)	2.477 (0.212)	2.458 (1413.6)	2.739 (0.384)
40	2.602 (136.7)	2.592 (0.163)	2.155 (170.4)	2.145 (0.150)	2.134 (183.2)	2.325 (0.175)
50	2.320 (0.135)	2.314 (0.112)	1.924 (61.75)	1.919 (0.117)	1.916 (81.20)	2.053 (0.123)
60	2.117 (0.103)	2.112 (0.089)	1.755 (16.656)	1.753 (0.096)	1.755 (22.07)	1.861 (0.099)
70	1.959 (0.081)	1.954 (0.072)	1.624 (0.588)	1.862 (0.218)	1.630 (1.318)	1.712 (0.081)
80	1.832 (0.069)	1.828 (0.063)	1.521 (0.071)	1.378 (0.129)	1.531 (0.066)	1.598 (0.069)

Results shown on logarithmic scale
GLS generalised least squares, GEE generalised estimating equations

Table 2: Median width (S.D.) of confidence intervals of the standard binary and augmented binary methods for the difference in response probabilities treatment effect

	Standard binary		Augmented binary (GLS)		Augmented binary (GEE)	
N	Unadjusted	Adjusted	Unadjusted	Adjusted	Unadjusted	Adjusted
30	0.675 (0.044)	0.686 (0.037)	0.567 (248.8)	0.554 (0.045)	0.554 (245.1)	0.614 (0.142)
40	0.592 (0.030)	0.597 (0.026)	0.492 (36.558)	0.464 (0.062)	0.488 (39.049)	0.528 (0.062)
50	0.536 (0.023)	0.539 (0.021)	0.442 (14.154)	0.470 (0.059)	0.442 (18.558)	0.472 (0.033)
60	0.490 (0.018)	0.492 (0.017)	0.406 (3.985)	0.403 (0.021)	0.407 (5.229)	0.429 (0.024)
70	0.455 (0.015)	0.456 (0.014)	0.377 (0.142)	0.375 (0.018)	0.379 (0.319)	0.396 (0.020)
80	0.426 (0.013)	0.428 (0.012)	0.354 (0.016)	0.351 (0.016)	0.357 (0.017)	0.371 (0.017)

Results shown on probability scale
GLS generalised least squares, GEE generalised estimating equations

Table 3: Average treatment effect (S.D.) in subsamples using the standard binary and augmented binary methods for the log-odds treatment effect

	Standard binary		Augmented binary (GLS)		Augmented binary (GEE)	
N	Unadjusted	Adjusted	Unadjusted	Adjusted	Unadjusted	Adjusted
30	0.606 (1.096)	0.526 (0.737)	0.783 (0.649)	0.747 (0.619)	0.812 (0.678)	0.773 (0.646)
40	0.595 (0.745)	0.543 (0.634)	0.794 (0.544)	0.765 (0.525)	0.828 (0.565)	0.796 (0.545)
50	0.572 (0.587)	0.536 (0.547)	0.790 (0.478)	0.767 (0.465)	0.821 (0.495)	0.795 (0.480)
60	0.570 (0.541)	0.540 (0.510)	0.788 (0.435)	0.770 (0.425)	0.816 (0.449)	0.795 (0.438)
70	0.577 (0.476)	0.551 (0.453)	0.794 (0.394)	0.902 (0.456)	0.821 (0.406)	0.802 (0.398)
80	0.568 (0.455)	0.546 (0.436)	0.790 (0.367)	0.707 (0.333)	0.817 (0.377)	0.801 (0.370)

Results shown on logarithmic scale
GLS generalised least squares, GEE generalised estimating equations

Table 4: Average treatment effect (S.D.) in subsamples using the standard binary and augmented binary methods for the difference in response probabilities treatment effect

	Standard binary		Augmented binary (GLS)		Augmented binary (GEE)	
N	Unadjusted	Adjusted	Unadjusted	Adjusted	Unadjusted	Adjusted
30	0.129 (0.178)	0.118 (0.163)	0.180 (0.145)	0.170 (0.137)	0.179 (0.153)	0.168 (0.143)
40	0.133 (0.153)	0.124 (0.142)	0.185 (0.124)	0.171 (0.116)	0.191 (0.128)	0.182 (0.123)
50	0.131 (0.132)	0.124 (0.125)	0.186 (0.110)	0.195 (0.117)	0.193 (0.113)	0.185 (0.109)
60	0.131 (0.123)	0.125 (0.117)	0.187 (0.101)	0.181 (0.098)	0.193 (0.104)	0.186 (0.100)
70	0.134 (0.109)	0.129 (0.104)	0.189 (0.091)	0.184 (0.089)	0.195 (0.094)	0.190 (0.091)
80	0.133 (0.104)	0.128 (0.101)	0.189 (0.085)	0.184 (0.083)	0.195 (0.087)	0.190 (0.085)

Results shown on probability scale
GLS generalised least squares, GEE generalised estimating equations

Table 5: Average treatment effect (S.D.) in permuted subsamples using the standard binary and augmented binary methods for the log-odds treatment effect

	Standard binary		Augmented binary (GLS)		Augmented binary (GEE)	
N	Unadjusted	Adjusted	Unadjusted	Adjusted	Unadjusted	Adjusted
30	0.011 (1.171)	0.005 (0.742)	0.001 (0.665)	0.049 (0.641)	-0.002 (0.673)	-0.002 (0.646)
40	0.012 (0.755)	0.006 (0.645)	-0.005 (0.559)	0.000 (0.537)	0.014 (0.583)	0.002 (0.555)
50	0.000 (0.608)	0.004 (0.575)	0.007 (0.487)	-0.003 (0.475)	-0.003 (0.512)	0.012 (0.492)
60	-0.005 (0.557)	0.004 (0.527)	-0.003 (0.449)	0.000 (0.437)	-0.004 (0.461)	-0.005 (0.452)
70	-0.004 (0.507)	0.001 (0.411)	0.004 (0.413)	0.000 (0.487)	-0.004 (0.424)	-0.007 (0.417)
80	0.005 (0.467)	-0.009 (0.457)	0.005 (0.390)	-0.002 (0.354)	0.000 (0.401)	-0.001 (0.386)

Results shown on logarithmic scale
GLS generalised least squares, GEE generalised estimating equations

Table 6: Average treatment effect (S.D.) in permuted subsamples using the standard binary and augmented binary methods for the difference in response probabilities treatment effect

	Standard binary		Augmented binary (GLS)		Augmented binary (GEE)	
N	Unadjusted	Adjusted	Unadjusted	Adjusted	Unadjusted	Adjusted
30	-0.003 (0.182)	-0.002 (0.166)	0.001 (0.151)	0.000 (0.144)	-0.002 (0.154)	0.000 (0.145)
40	0.001 (0.156)	0.000 (0.148)	0.001 (0.130)	-0.002 (0.123)	-0.004 (0.136)	0.000 (0.130)
50	-0.001 (0.142)	-0.002 (0.134)	0.002 (0.117)	0.000 (0.112)	0.001 (0.121)	0.000 (0.118)
60	0.000 (0.129)	-0.002 (0.121)	0.002 (0.106)	-0.003 (0.104)	-0.002 (0.113)	0.001 (0.108)
70	-0.004 (0.120)	0.000 (0.114)	0.001 (0.101)	0.001 (0.098)	0.001 (0.104)	-0.001 (0.101)
80	-0.002 (0.112)	0.000 (0.108)	0.001 (0.094)	0.000 (0.092)	0.000 (0.097)	-0.001 (0.094)

Results shown on probability scale
GLS generalised least squares, GEE generalised estimating equations
